# Molecular Surveillance of Low Pathogenic Avian Influenza Viruses in Wild Birds across the United States: Inferences from the Hemagglutinin Gene

**DOI:** 10.1371/journal.pone.0050834

**Published:** 2012-12-04

**Authors:** Antoinette J. Piaggio, Susan A. Shriner, Kaci K. VanDalen, Alan B. Franklin, Theodore D. Anderson, Sergios-Orestis Kolokotronis

**Affiliations:** 1 National Wildlife Research Center, Wildlife Services, United States Department of Agriculture, Fort Collins, Colorado, United States of America; 2 Sackler Institute for Comparative Genomics, American Museum of Natural History, New York, New York, United States of America and Department of Biological Sciences, Fordham University, Bronx, New York, United States of America; Erasmus Medical Center, The Netherlands

## Abstract

A United States interagency avian influenza surveillance plan was initiated in 2006 for early detection of highly pathogenic avian influenza viruses (HPAIV) in wild birds. The plan included a variety of wild bird sampling strategies including the testing of fecal samples from aquatic areas throughout the United States from April 2006 through December 2007. Although HPAIV was not detected through this surveillance effort we were able to obtain 759 fecal samples that were positive for low pathogenic avian influenza virus (LPAIV). We used 136 DNA sequences obtained from these samples along with samples from a public influenza sequence database for a phylogenetic assessment of hemagglutinin (HA) diversity in the United States. We analyzed sequences from all HA subtypes except H5, H7, H14 and H15 to examine genetic variation, exchange between Eurasia and North America, and geographic distribution of LPAIV in wild birds in the United States. This study confirms intercontinental exchange of some HA subtypes (including a newly documented H9 exchange event), as well as identifies subtypes that do not regularly experience intercontinental gene flow but have been circulating and evolving in North America for at least the past 20 years. These HA subtypes have high levels of genetic diversity with many lineages co-circulating within the wild birds of North America. The surveillance effort that provided these samples demonstrates that such efforts, albeit labor-intensive, provide important information about the ecology of LPAIV circulating in North America.

## Introduction

Waterfowl and shorebirds are regarded as natural reservoirs for avian influenza A viruses (AIV) [1], [2]. Many species of waterfowl have the potential to shed high quantities of AIV in their feces, which can then be transmitted to other individuals when these birds congregate in large numbers in aquatic environments [1], [3], [4]. While clinical signs are generally absent in wild bird hosts infected with AIV, these viruses are of interest to agricultural operations because they can cause disease and loss of production in poultry.

Influenza subtypes are defined by the antigenicity of the hemagglutinin (HA) and neuraminidase (NA) envelope proteins [1]. Sixteen HA antigens (H1–H16) and nine NA antigens (N1–N9) have been isolated from at least 12 orders of wild birds [5]–[8]. Two virulence phenotypes have been described for AIV based on their pathogenicity in poultry: low pathogenic avian influenza viruses (LPAIV) and highly pathogenic avian influenza viruses (HPAIV) [6]. To date, only H5 and H7 subtypes have been responsible for HPAIV phenotypes in birds [6]. Since 1997 Southeast Asian strains of HPAIV H5N1 have been responsible for high mortality in domestic poultry, as well as causing severe disease in some wild birds and mammals, including humans [9]. The westward expansion of Asian strain HPAIV H5N1 brought renewed attention to the threat that AIV pose to wildlife, livestock, and human health.

**Figure 1:**
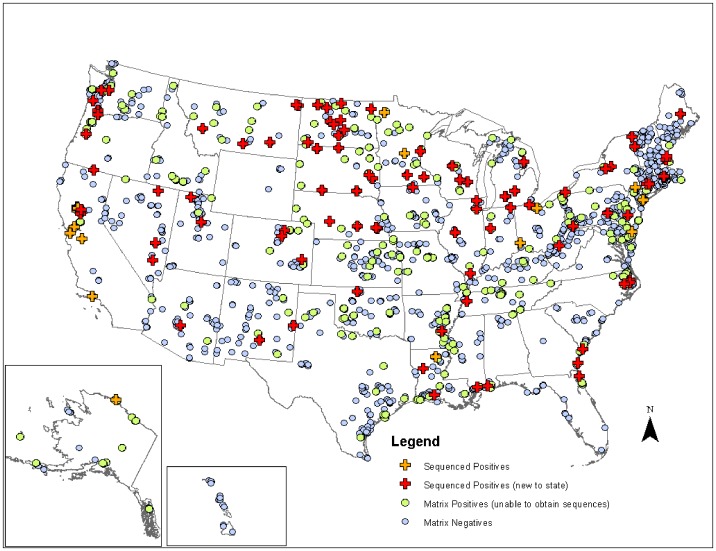
Geographic locations of avian fecal samples collected in the United States and HA sequences generated during USDA surveillance efforts from 2006 through 2007. This map shows the total avian fecal samples collected across the United States and shows which of those samples were matrix positive and matrix negative. Of the matrix positive samples that we successfully sequenced we also show sampling localities and differentiate between sequences representing HA subtypes that have been previously documented in that state (HA sequences from the same subtype and state also found on NCBI Influenza Virus Resource during our searches) and those that were newly documented in that state through this study.

Wild birds have been implicated in the global spread of Asian strain HPAIV H5N1 [10]–[12]. Consequently, a United States interagency avian influenza surveillance plan (hereafter, surveillance) was initiated for early detection of the virus [13]. Surveillance focused on a variety of sampling strategies to screen wild birds from each of the 50 states for the presence of AIV [14]–[16]. One of the strategies was the testing of wild bird fecal samples from aquatic areas throughout the U.S from April 2006 through December 2007 [14]. While a primary goal of this surveillance effort was early detection of Asian strain HPAIV H5N1 in the U.S., a secondary goal was to characterize LPAIV subtypes circulating in the U.S., assess phylogenetic relationships within each sampled subtype, and investigate potential intercontinental gene transfer [12], [17]–[29]. By combining our wild bird fecal surveillance HA sequences with avian (domestic and wild bird) derived HA gene sequences from a publicly available database we aimed 1) to assess genetic variation within each of the sequenced HA subtypes, 2) to quantify the extent of putative HA gene flow across continents, especially HA gene flow from Eurasia in to North America and 3) to examine geographic distribution of HA subtypes across North America.

**Figure 2:**
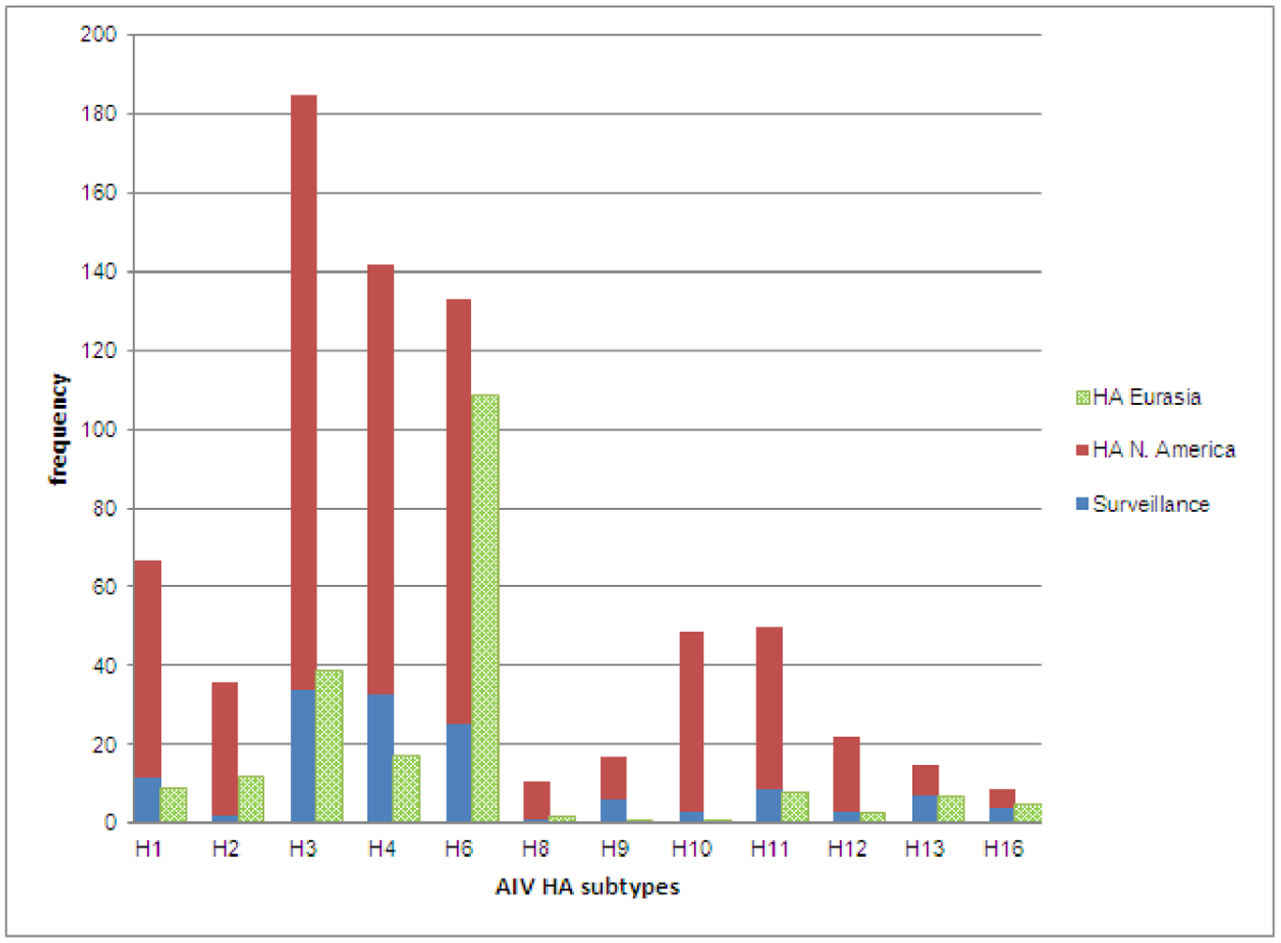
Numbers of sequences used in this study showing frequency of each subtype per continent and number surveillance sequences relative to sequences obtained from NCBI Influenza Virus Resource.

**Table 1:** Summary statistics for all HA subtypes sampled during the United States interagency surveillance effort along with other North American, Asian, and European subtypes downloaded from NCBI Influenza Virus Resource.

Subtype	Region	*n*	*n*_USDA_	Length (bp)	*h*	*H_d_*	*S*	*θ*_W_	*F*_S_	*K*_ST_
**H1**		94	12	1698	88	0.999	751	0.08952	−10.163*	0.09449***
	N. America	85			80	0.999	696	0.08248	−9.806*	
	Eurasia	9			9	1.0	531	0.11891	1.528	
**H2**		46	2	1686	42	0.996	576	0.07801	−0.607	0.37203***
	N. America	34			30	0.993	541	0.07876	0.253	
	Eurasia	12			12	1.0	217	0.04262	−0.144	
**H3**		190	34	1689	181	0.999	794	0.08327	−33.353**	0.18928***
	N. America	151			143	0.998	750	0.08174	−29.281***	
	Eurasia	39			39	1.0	473	0.06636	−5.457*	
**H4**		126	33	1692	119	0.998	727	0.10873	−21.977**	0.25032***
	N. America	109			102	0.998	638	0.08890	−21.511**	
	Asia	17			17	1.0	422	0.08073	−0.654	
**H6**		217	25	1704	207	1.0	874	0.12884	−65.782***	0.11176***
	N. America	108			102	0.999	734	0.11026	−12.045*	
	Europe	8			8	1.0	258	0.06093	0.873	
	Asia	101			97	0.999	676	0.09551	−24.304***	
**H8**		12	1	1698	12	1.0	355	0.07001	0.197	0.04479
	N. America	10			10	1.0	185	0.03895	0.204	
	Asia	2			2	1.0	203	0.11955	5.313	
**H9**		238	6	1680	232	1.0	883	0.09494	−106.984***	0.1047***
	N. America	24			24	1.0	492	0.0844	−1.381	
	Eurasia	209			206	1.0	901	0.09382	−100.54***	
**H10**		51	3	1683	49	0.998	558	0.08926	−6.020*	0.30163***
	N. America	46			44	0.998	365	0.05366	−9.036*	
	Eurasia	5			5	1.0	190	0.05476	2.162	
**H11**		49	9	1695	49	1.0	636	0.10236	−7.487*	0.38747***
	N. America	41			41	1.0	459	0.07065	−10.015**	
	Asia	8			8	1.0	203	0.04642	1.001	
**H12**		22	3	1692	22	1.0	504	0.09360	−1.24	0.40124*
	N. America	19			19	1.0	293	0.05252	−1.59	
	Eurasia	3			3	1.0	114	0.04573	3.223	
**H13**		37	7	1698	36	0.998	659	0.09521	−0.864	0.15442***
	N. America	18			17	0.993	552	0.09679	1.704	
	Eurasia	19			19	1.0	543	0.09166	−0.07	
**H16**		10	4	1698	10	1.0	548	0.13663	1.559	0.05997
	N. America	5			5	1.0	372	0.11319	2.939	
	Europe	5			5	1.0	432	0.13504	3.184	

*n*, number of individual sequences; *h*, number of haplotypes; *H*_d_, haplotype diversity; *S*, number of polymorphic sites; *θ*_W_, Watterson’s estimate of nucleotide diversity; *F*_S_, Fu’s *F*_S_; *K*_ST_, genetic differentiation.

*** , *p*≤0.001; **, *p*≤0.006; *, *p*≤0.05.

## Materials and Methods

### Ethics Statement

Full details of this study were approved by a USDA/APHIS/WS/National Wildlife Research Center review and complied with the institutional animal care and use policies. No permits were required for field collection of avian fecal samples used in this study, which were collected from public lands, did not require specific permission for access, and no wild animals, specifically endangered species, were affected.

**Figure 3:**
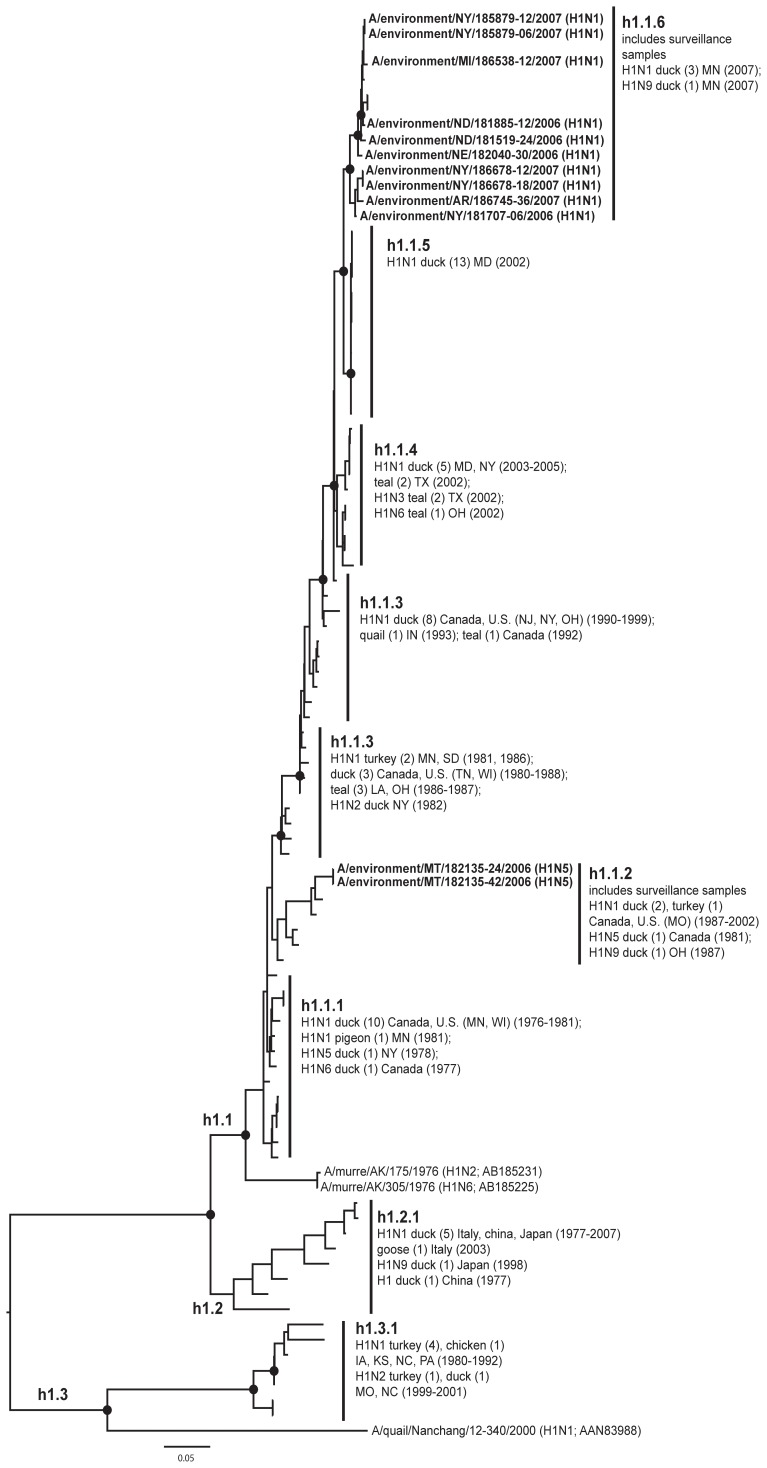
ML phylogram of H1 sequences from North America and Eurasia including U.S. surveillance samples. ML phylogram of influenza A subtype H1. Branch lengths represent genetic distance. Dots show nodes with significant bootstrap support. USDA surveillance sequences from avian fecal samples are shown in bold.

**Figure 4:**
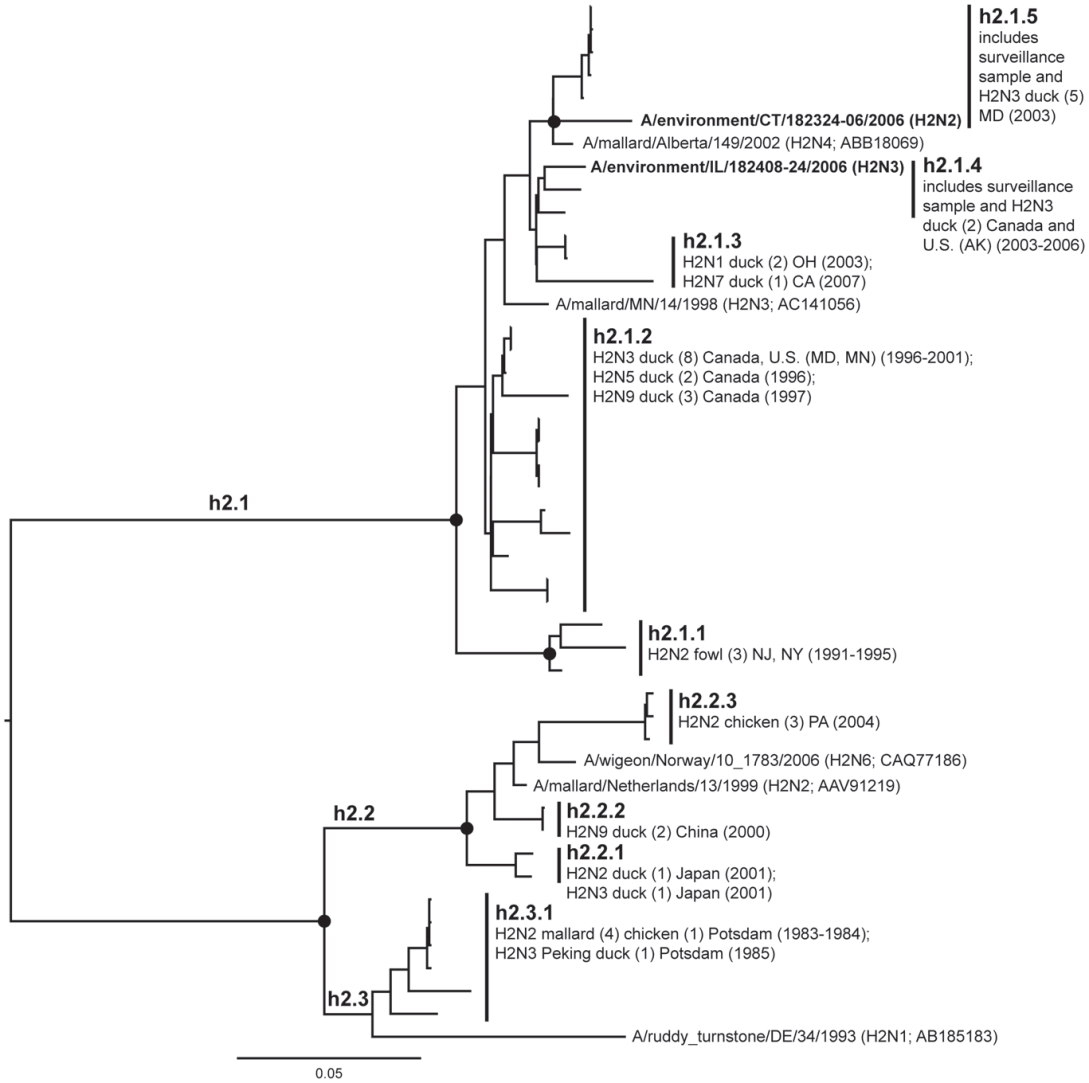
ML phylogram of H2 sequences from North America and Eurasia including U.S. surveillance samples. ML phylogram of influenza A subtype H2. Branch lengths represent genetic distance. Dots show nodes with significant bootstrap support. USDA surveillance sequences from avian fecal samples are shown in bold.

### Surveillance Sampling

Approximately 1000 wild bird fecal samples per U.S. state were collected in 2006 for a total of 50,184 samples. In 2007, a sampling scheme based on a weighted allocation of key flyway states and regions that had high recovery in 2006 was used and 25,348 additional samples were obtained [15], [30]. Fresh wild bird feces were collected on Dacron swabs and stored in BA-1 liquid media (Hanks' M-199 salts, 1% bovine serum albumin, 350 mg/L of sodium bicarbonate, 100 U/mL of penicillin, 100 mg/L of streptomycin, and 1 mg/L of fungizone in 0.05 M Tris, pH 7.6) and kept chilled until shipment. Samples were generally shipped overnight to the National Wildlife Research Center, Fort Collins, CO, (NWRC) within 24 hours of collection.

**Figure 5:**
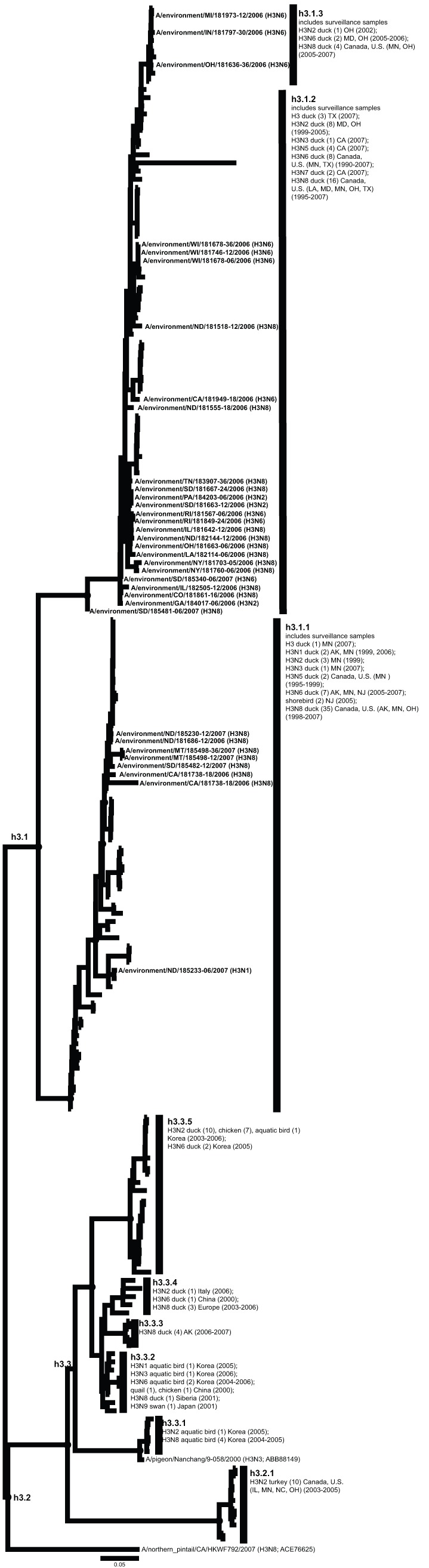
ML phylogram of H3 sequences from North America and Eurasia including U.S. surveillance samples. ML phylogram of influenza A subtype H3. Branch lengths represent genetic distance. Dots show nodes with significant bootstrap support. USDA surveillance sequences from avian fecal samples are shown in bold.

**Figure 6:**
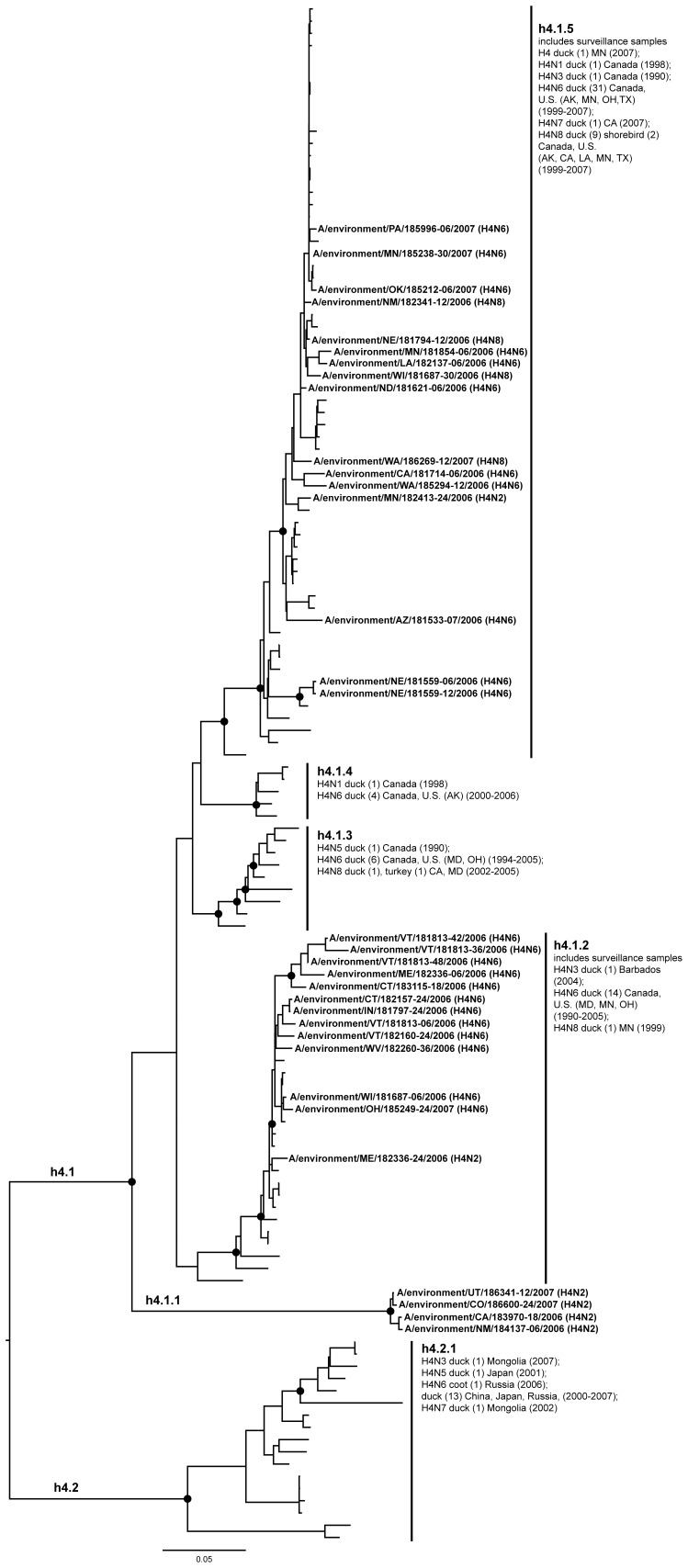
ML phylogram of H4 sequences from North America and Eurasia including U.S. surveillance samples. ML phylogram of influenza A subtype H4. Branch lengths represent genetic distance. Dots show nodes with significant bootstrap support. USDA surveillance sequences from avian fecal samples are shown in bold.

### RT-PCR, Virus Isolation, Subtyping, and Sequencing

On arrival at NWRC, up to five fecal samples were pooled based on collection locality. Within 48 hours of collection, pooled samples were initially tested by real-time reverse transcriptase polymerase chain reaction (RRT-PCR) for AIV RNA detection. RNA was extracted using a King Fisher 96 magnetic particle processor (Thermo Fisher Scientific, Waltham, MA) with Ambion MagMAX 96 AI/ND Viral RNA Isolation Kits (Life Technologies, Carlsbad, CA) or a BioRobot MDx Workstation with a modified QIAamp viral RNA kit (Qiagen, Valencia, CA). Primers and probe specific for the influenza A matrix gene [31] were used in conjunction with the ABI one-step RT-PCR master mix and the ABI 7900 Real Time PCR system (Life Technologies Corp, Carlsbad, CA) with thermocycler conditions described in VanDalen et al. [32]. Samples from pools that tested positive were re-tested (this time individually) by RRT-PCR for confirmed presence of matrix gene as well as H5 and H7 genes [31]. All putative H5 and H7 RRT-PCR positive samples were sent to the National Veterinary Services Laboratory (NVSL), Ames, Iowa without further testing at NWRC. All other RRT-PCR matrix positive samples were inoculated into 11-day-old embryonated SPF chicken eggs [33] at the NWRC for virus isolation. Aliquots of each successful isolate were shipped to NVSL and subtyped using hemagglutination inhibition (HI) and neuraminidase inhibition (NI) tests. RNA was also extracted from isolates and conventional RT-PCR was performed using QIAGEN OneStep RT-PCR kits (Qiagen, Valencia, CA) following manufacturer’s protocol with annealing temperatures ranging from 52–55°C. To amplify each HA gene a suite of internal HA-specific (H1–H15) primers developed by the Southeast Poultry Research Laboratory (SEPRL), Athens, GA were used along with internal primers designed at the NWRC (H3, H6, H8, H9, H13, Table S1; H16 [34]). These internal primers were used with the HA end universal primers, Hgga+ and H-T7, also developed by SEPRL [35]. Some isolates could not be amplified using the H1–H15 specific primers and re-amplification was sometimes accomplished using only the HA end universal primers. RT-PCR products were purified using ExoSAP-IT (USB Corp., Cleveland, OH) and sequenced using ABI BigDye Terminator v3.1 chemistry. The products of the cycle sequencing reaction were purified using Millipore Multiscreen 96 well cleanup plates (Millipore, Billerica, MA) with G-50 Sephadex (GE Healthcare, Pittsburgh, PA). The purified sequencing product was visualized on an ABI 3130xl DNA Analyzer. Sequences were assembled and checked for errors using Sequencher v4.8 (Gene Codes Corp., Ann Arbor, MI).

**Figure 7:**
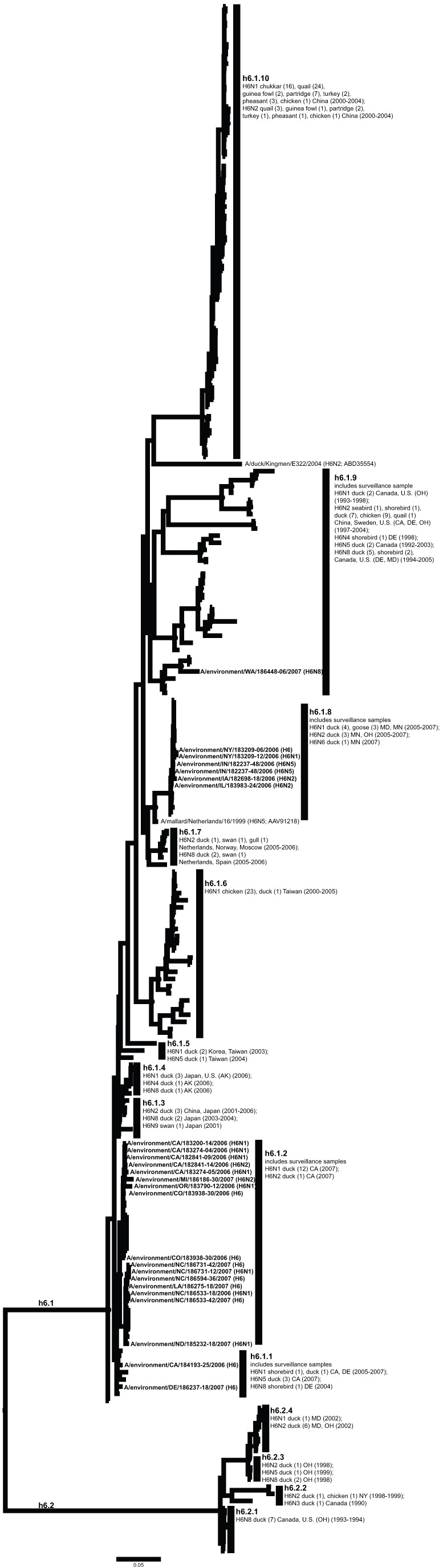
ML phylogram of H6 sequences from North America and Eurasia including U.S. surveillance samples. ML phylogram of influenza A subtype H6. Branch lengths represent genetic distance. Dots show nodes with significant bootstrap support.

**Figure 8:**
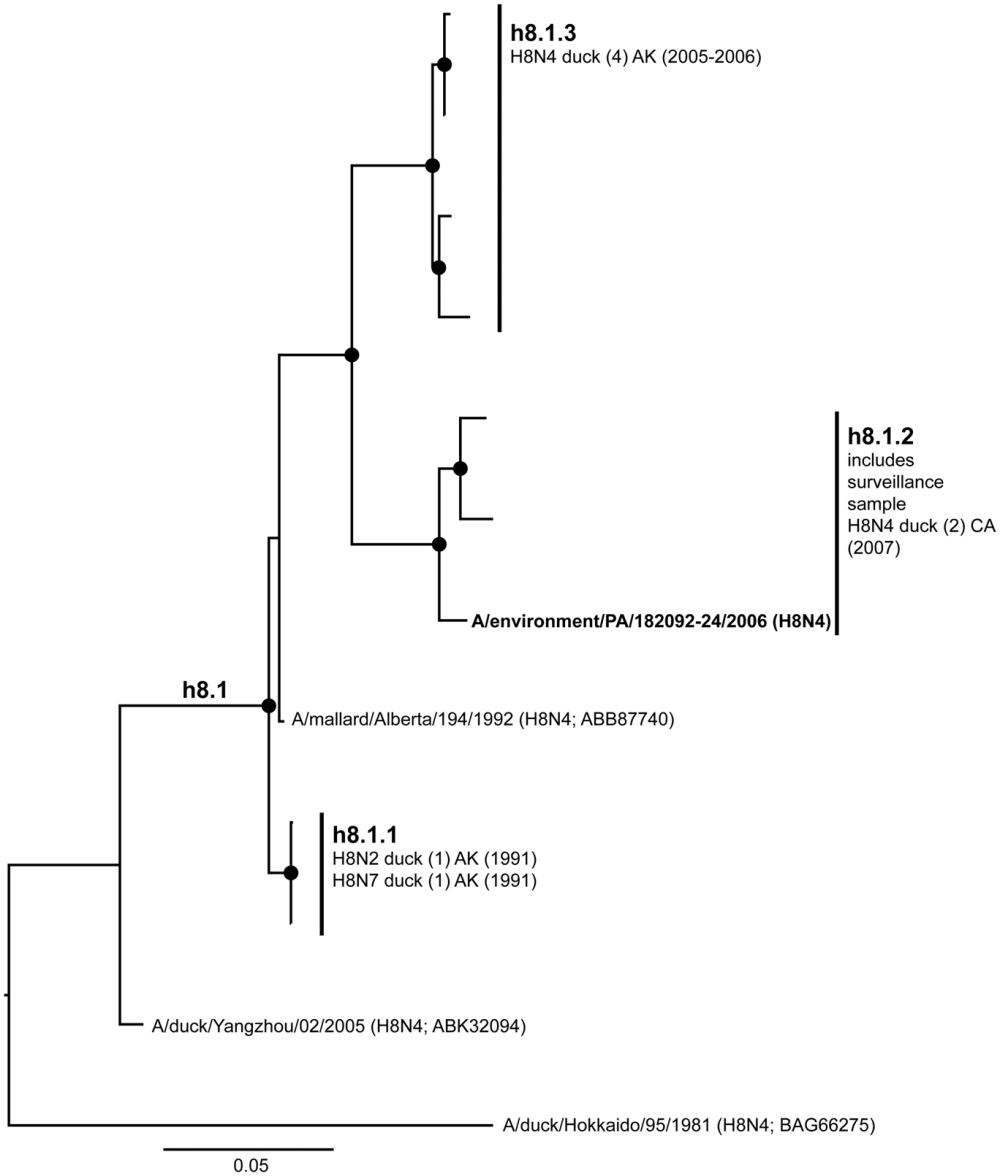
ML phylogram of H8 sequences from North America and Eurasia including U.S. surveillance samples. ML phylogram of influenza A subtype H8. Branch lengths represent genetic distance. Dots show nodes with significant bootstrap support. USDA surveillance sequences from avian fecal samples are shown in bold.

### Sequence and Evolutionary Analyses

We compiled a collection of HA sequence data from North America and Europe composed of a combination of sequenced samples from the surveillance effort and publicly available avian full-length HA sequences from the NCBI Influenza Virus Resource (http://www.ncbi.nlm.nih.gov/genomes/FLU/FLU.html). Coding sequences were aligned in MUSCLE v3.6-3.8.31 (Edgar, 2004) and inspected in Se-Al v2.0a11 (http://tree.bio.ed.ac.uk/software/seal). We focused on publicly available complete HA gene sequences from 1990–2008 from U.S., Canada, Europe and Asia for each subtype we sequenced from the surveillance samples (all HAs except H5, H7, H14 and H15). Later we added sequences from as far back as the 1960s for H1, H9, and H13 subtypes because of the limited availability of sequences for these subtypes. We tried to minimize our use of sequences that were >25 years old to avoid working with those that might have been based on isolates subjected to multiple passages through chicken eggs which has the potential to cause the accumulation of mutations [36]. In order to increase alignment quality, we excluded partial sequences, those with large deletions, and those with premature stop codons.

**Figure 9:**
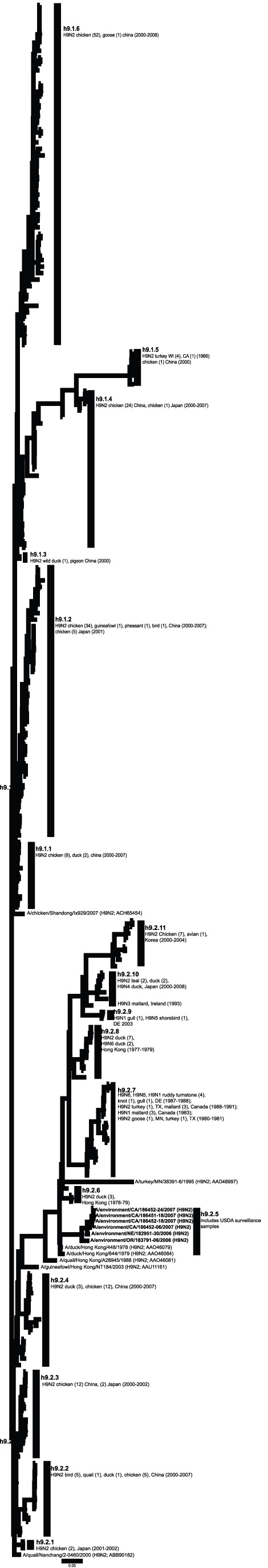
ML phylogram of H9 sequences from North America and Eurasia including U.S. surveillance samples. ML phylogram of influenza A subtype H9. Branch lengths represent genetic distance. Dots show nodes with significant bootstrap support. USDA surveillance sequences from avian fecal samples are shown in bold.

**Figure 10:**
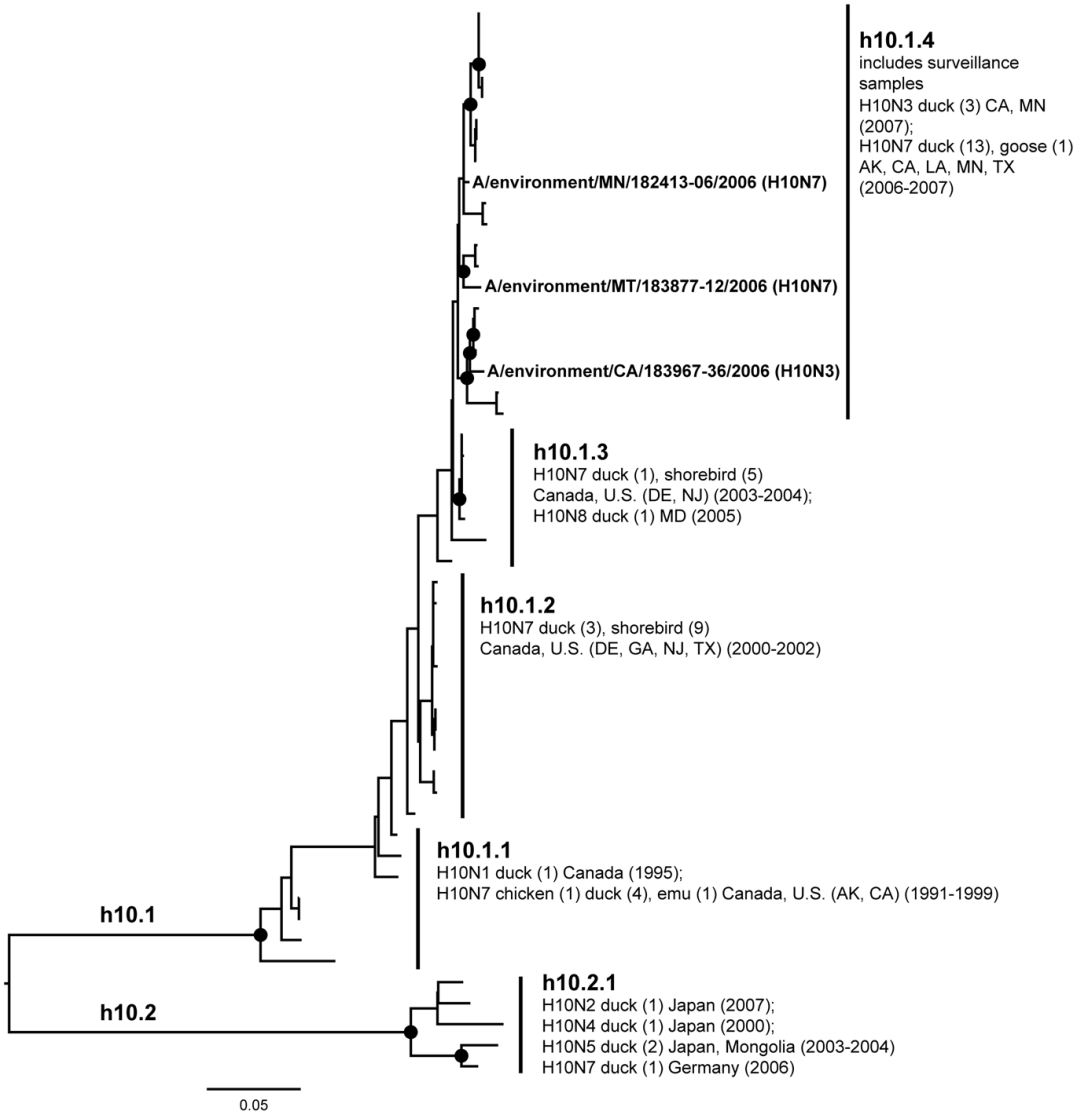
ML phylogram of H10 sequences from North America and Eurasia including U.S. surveillance samples. ML phylogram of influenza A subtype H10. Branch lengths represent genetic distance. Dots show nodes with significant bootstrap support. USDA surveillance sequences from avian fecal samples are shown in bold.

Nucleotide diversity was estimated per HA subtype using Watterson’s *θ*_W_ estimator [37] on a per-site basis. Summary statistics from sequence data also included number of haplotypes (*h*), haplotype diversity (*H_d_*), and polymorphic sites (*S*) [38]. We employed Fu’s *F*_S_ [39] test statistic to examine the conformity of our sample sequences site frequency spectrum to the neutral expectations. Fu’s *F*_S_ draws from the haplotype distribution and acquires large negative values in the presence of rare alleles [39]. Overall, the haplotype frequency distribution (Fu’s *F*_S_ test) can be a result of population size growth following a potential bottleneck as expected according to extensive simulation studies [39], [40]. Significance was estimated using 1000 Monte Carlo simulations under the neutral coalescent process [41] conditioning on the number of polymorphic sites and the absence of recombination. Genetic differentiation worldwide was quantified by *K*_ST_ [42] using 1000 permutations to assess statistical significance. Neutrality test statistics were not computed for different continents if the null hypothesis of panmixia was rejected by the genetic differentiation test. The above calculations were carried out in DnaSP v5.10 (http://www.ub.edu/dnasp) [43]. Descriptive statistics and correlations were computed in the R software environment (http://www.r-project.org) locally (R v2.10.1) and on www.wessa.net [44].

**Figure 11:**
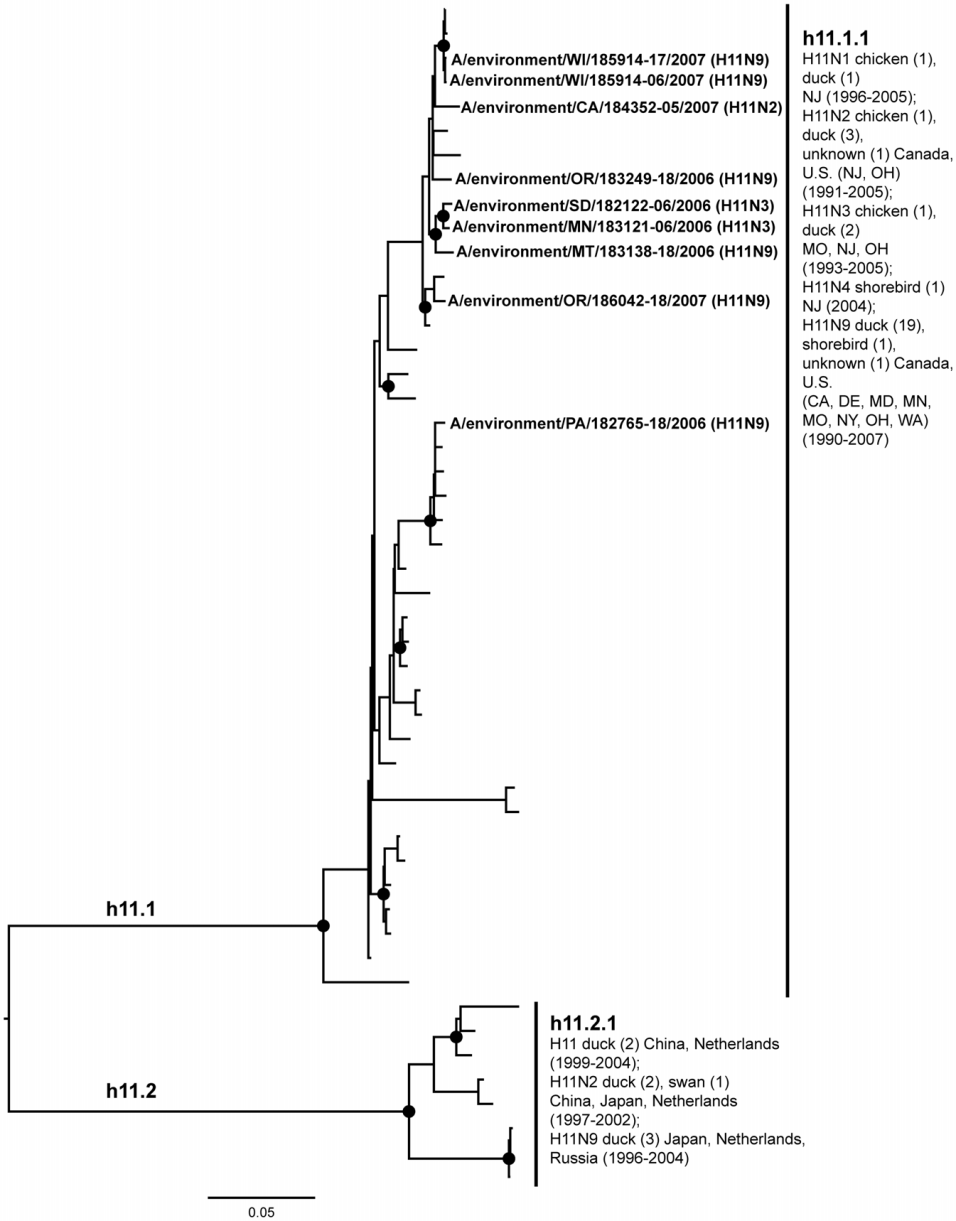
ML phylogram of H11 sequences from North America and Eurasia including U.S. surveillance samples. ML phylogram of influenza A subtype H11. Branch lengths represent genetic distance. Dots show nodes with significant bootstrap support. USDA surveillance sequences from avian fecal samples are shown in bold.

**Figure 12:**
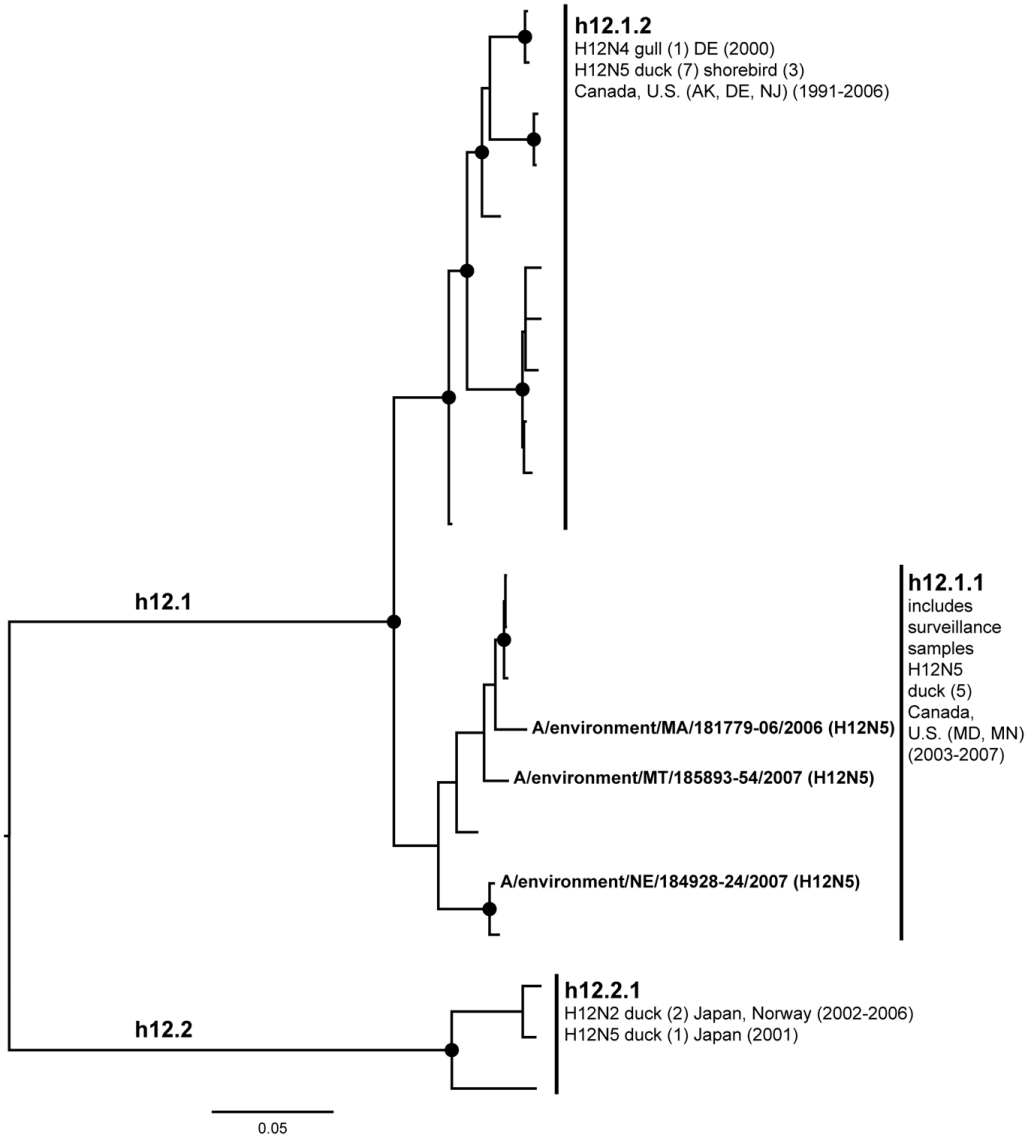
ML phylogram of H12 sequences from North America and Eurasia including U.S. surveillance samples. ML phylogram of influenza A subtype H12. Branch lengths represent genetic distance. Dots show nodes with significant bootstrap support. USDA surveillance sequences from avian fecal samples are shown in bold.

Phylogenetic analysis was accomplished in a maximum likelihood (ML) framework using the parallel Pthreads version of RAxML v7.1.0-7.2.6 [45], [46] on an 8-way shared memory (32GB RAM) server at the American Museum of Natural History. We implemented the general time-reversible (GTR) substitution model [47] accounting for among-site rate heterogeneity using the Γ distribution and four rate categories [48]. Node support was evaluated with 500 nonparametric bootstrap pseudoreplicates [49] and was summarized by filtering the best ML tree through the swarm of bootstrap trees. This did not provide a consensus tree but rather a tree showing the proportion of bootstrap trees that contained a given node.

**Figure 13:**
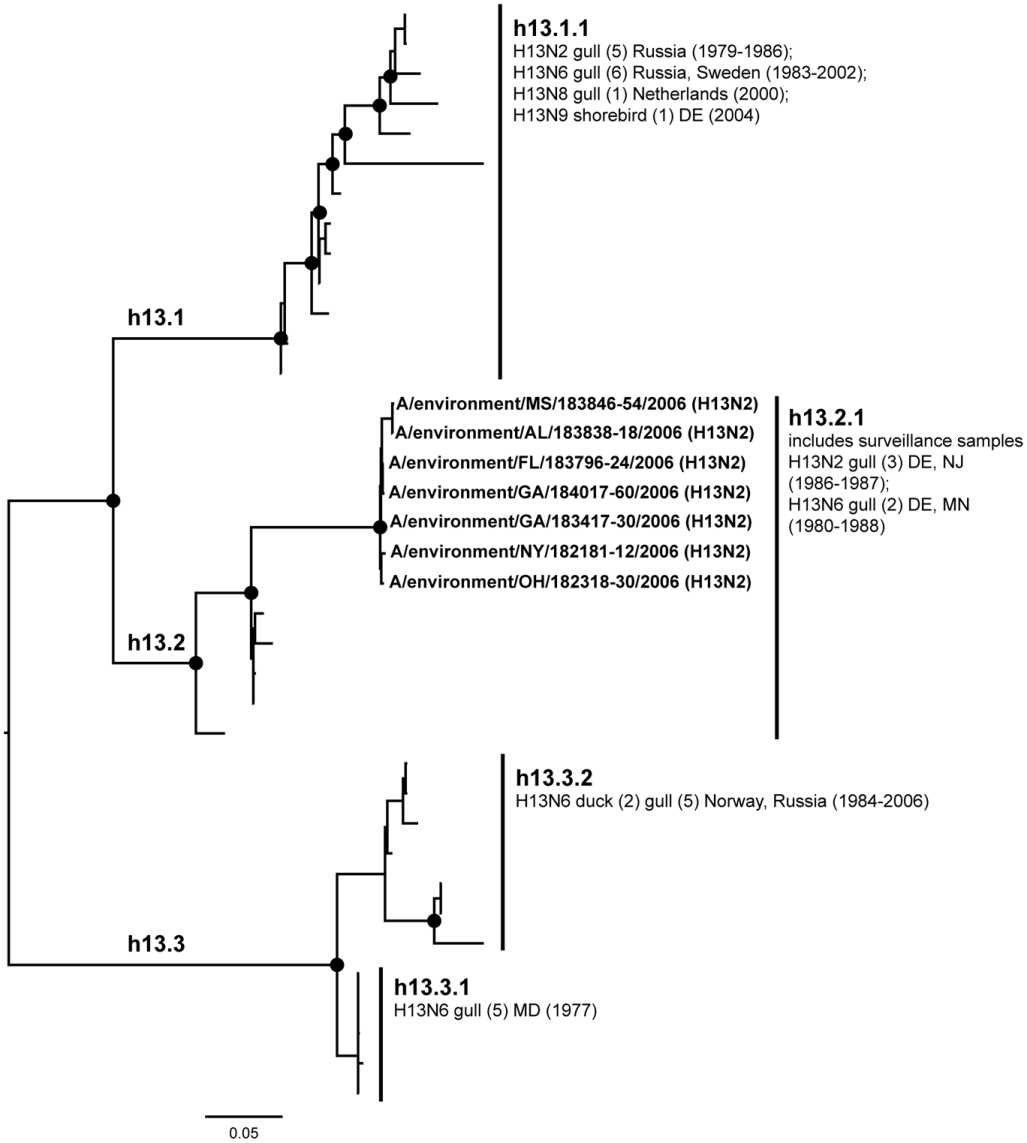
ML phylogram of H13 sequences from North America and Eurasia including U.S. surveillance samples. ML phylogram of influenza A subtype H13. Branch lengths represent genetic distance. Dots show nodes with significant bootstrap support. USDA surveillance sequences from avian fecal samples are shown in bold.

**Figure 14:**
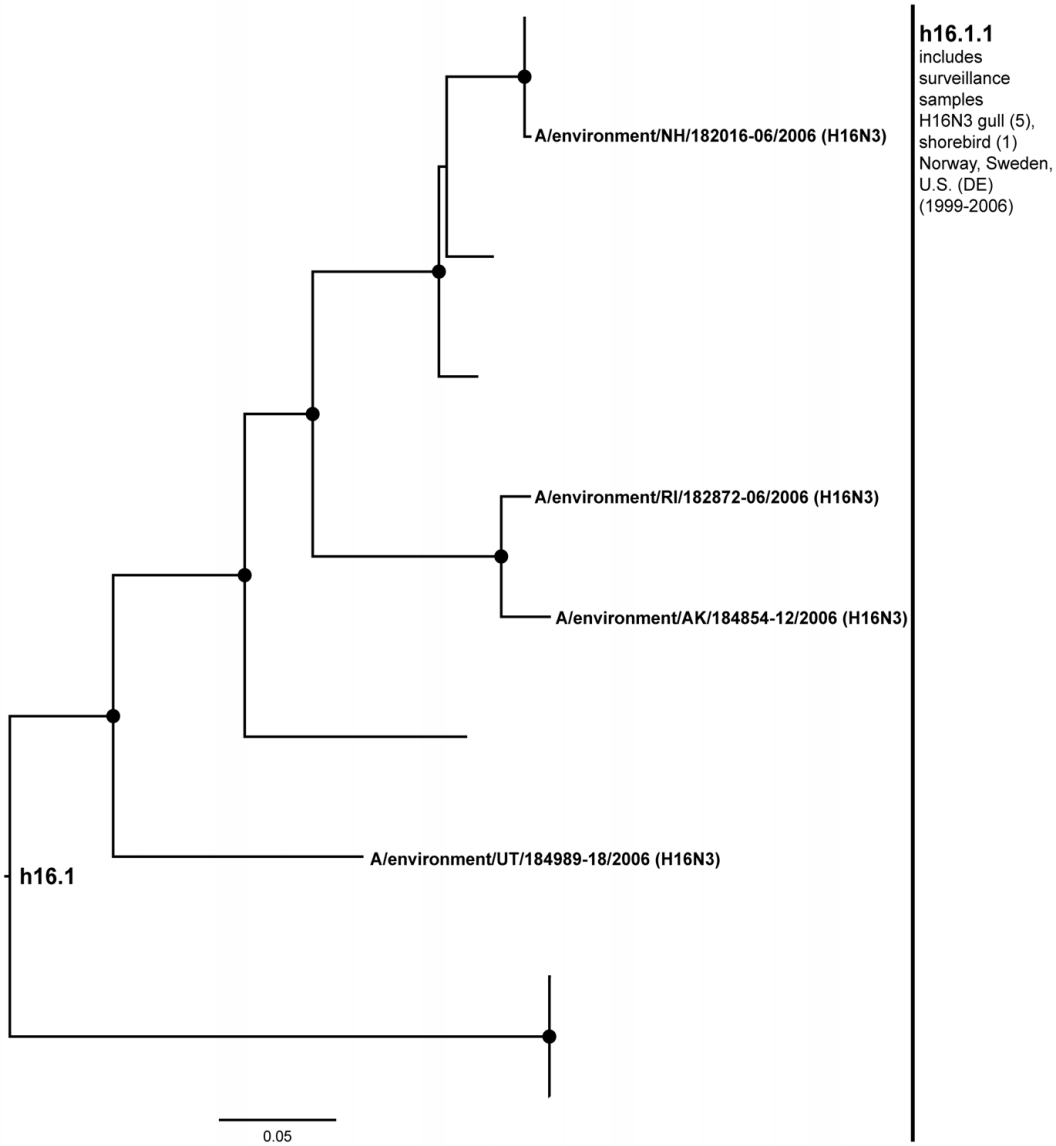
ML phylogram of H16 sequences from North America and Eurasia including U.S. surveillance samples. ML phylogram of influenza A subtype H16. Branch lengths represent genetic distance. Dots show nodes with significant bootstrap support. USDA surveillance sequences from avian fecal samples are shown in bold.

## Results and Discussion

Across both years of sampling 16,538 wild bird fecal sample pools representing 75,532 individual samples were tested via RRT-PCR. A total of 759 influenza A matrix gene RRT-PCR positives were identified, 214 of which yielded AIV isolates suitable for subtyping by HI. Subtypes identified included H1–H4, H6–H14, and H16. Subtypes H5 and H7 are not represented due to the samples potential pathogenicity; any H5 or H7 positives were submitted to NVSL. We did not detect any H15 subtypes among surveillance samples. From these isolates we generated 136 HA sequences from across the U.S.; GenBank numbers are provided in Supporting Information (Table S2). Most HA sequences corresponded with the HI subtype determination with the exception of one sample identified as H14 by HI test that we could not amplify. There were also four samples identified as H7 by HI test (matrix gene positive but not identified as H7 until HI test), but when we amplified and sequenced these samples they most closely matched H16 sequences from the NCBI Influenza Virus Resource [34]. Although surveillance sequences were a small proportion of total sequences in our alignments (Table 1), the 136 sequenced HA genes represented circulating LPAIV in wild birds across the U.S. and include HA subtypes in areas where they had not been previously documented (Figure 1) and unique genetic lineages of some subtypes (H1, H3, H4, H9, H13, H16). When the surveillance sequences were combined with sequences from the public database that met our parameters both North America and Eurasia were represented (Figure 2).

Our primary goal in this study was to advance our understanding of HA geographic distributions and HA gene diversity circulating in wild bird populations in the U.S. through surveillance efforts, DNA sequencing, AI sequence database mining, and phylogenetic analysis. The ML phylogenetic trees of each subtype (Figures 3, 4, 5, 6, 7, 8, 9, 10, 11, 12, 13, 14) show that the HA sequences from the U.S. wild bird fecal surveillance effort consistently clustered with other North American HA sequences for all HA subtypes except H9. Overall, our phylogenetic analyses confirmed previously identified instances of intercontinental exchange and added new evidence of exchange but also demonstrated that some HA subtypes are well established in North America, without recent evidence of exchange [12], [17]–[20], [22]–[27]. Our results are unique because they focus on 1) solely avian isolates, 2) continental transfer within the past 20 years (in some cases 40 years), and 3) the addition of sequences obtained from extensive U.S. surveillance efforts.

### Intercontinental Exchange and U.S. Geographic Distribution of Wild Bird HA Genes

Documenting evidence for intercontinental exchange of AIV provides important information on the likelihood that wild birds may transfer AIV strains that pose a threat to agriculture and public health between continents. Our analysis identified AIV HA subtypes with patterns of recurring (within the past 20 years or in the case of H1, H9 and H13, the past 40 years) exchange between North America and Europe and/or Asia (H1, H2, H3, H6, H9, H13, and H16). In contrast, HA gene sequences representing H4, H8, H10, H11, and H12 subtypes appear to be well established in North America, without recent evidence of intercontinental exchange. These subtypes harbor diverse lineages unique to North America suggesting evolution has occurred within this continental system.

In the case of H1 (Figure 3; h1.1 and h1.3), North American sequences formed paraphyletic groups [24]. The North American lineage (h1.1) exhibited a temporal progression of genetic diversity from the early 1970s to 2007 (h1.1). The surveillance sequences fell into this lineage (h1.1.2 and h1.1.6) and some formed previously unidentified subclades. Some of these sequences also came from states where H1 was not represented in the public AIV sequence database during our search (Arkansas, North Dakota, Nebraska, Michigan, and Montana). There was a second lineage of North American H1 sequences that are largely from domestic birds (h1.3.1) that was more closely related to Eurasian sequences from wild and domestic birds (for this and all analyses, it is unknown if Asian duck samples are wild or domestic) than to the primary North American lineage as has been noted previously [24]. This clade of domestic bird sequences suggested that H1 exchange within this lineage was mediated within an agricultural setting and may not be associated with exchange with wild birds. Eurasian avian sequences formed a separate monophyletic group (h1.2.1). In the H1 North American clade where most of the published AIV H1 sequences fell (h1.1), most subtypes were H1N1, but our surveillance uncovered H1N5 in Montana in 2006. This subtype had been previously sequenced in a mallard in New York in 1978 (h1.1.1) and a pintail duck in Alberta in 1981 (h1.1.2).

H2 sequences formed two major lineages and each included domestic and wild birds (Figure 4), with one a purely North American lineage (h2.1) and the second indicating exchanges among North America and Eurasia (h2.2 and h2.3); a relationship that has been previously documented [26]. One case (h2.2) suggested a jump (no earlier than 1999) from Eurasian wildfowl to chickens in the northeastern U.S (h2.2.3). Our surveillance only successfully sequenced two H2 samples (h2.1.4 and h2.1.5).

H3 was the most common subtype sequenced from the surveillance samples (N = 34, Figure 2). Some of these H3 sequences belonged to previously unidentified subclades (Figure 5; h3.1.1 and h3.1.2) and were collected from 13 states that were not previously represented in available H3 sequence data from the past 20 years (Colorado, Georgia, Illinois, Indiana, Michigan, Montana, Nevada, North Dakota, Pennsylvania, Rhode Island, South Dakota, Tennessee, and Wisconsin). North American H3 sequences fell into three clades. The largest clade overall was made up of wild bird H3 sequences from across North America, including the surveillance sequences (h3.1). A smaller clade of U.S. domestic bird samples was most closely related to a single wild bird from California (h3.2). Together these were sister to a predominantly Eurasian clade, which included both wild and domestic birds (h3.3). This Eurasian clade also included Alaskan wild bird sequences (h3.3.3) that were closely related to European duck sequences (h3.3.4), suggesting that H3 subtypes have been shared across continents within the past 10 years. We also identified two distinct transfer events in the past decade for H3: one between North American domestic birds (h3.2.1) and Asian wild birds (h3.3.1) and another between Eurasian wild and domestic birds (h3.3.2 and h3.3.4) and Alaskan wild birds (h3.3.3) as previously identified (Koehler et al. 2008; Lee et al. 2011). However, it is important to note that non-avian species may have mediated this exchange (Liu et al. 2009; Shi et al. 2010) as is possible across most influenza A subtypes.

We found the most pronounced pattern of intercontinental exchange in H6, as in previous phylogenetic analyses of AIV HA genes [12], [27], [50]. Within one clade (h6.1), there was a mixture of sequences from North American and Eurasian wild and domestic birds with multiple exchange events evident (Figure 7). Surveillance sequences fell into this clade and added some new subclades (h6.1.2 and h6.1.8) and samples from 11 states where H6 sequences had not been documented in the past 20 years (Colorado, Iowa, Illinois, Indiana, Louisiana, Michigan, North Carolina, North Dakota, New York, Oregon, Washington). A second clade (h6.2) was made up exclusively of North American samples (wild and domestic birds). This clade showed a clear temporal progression in wild birds: after an initial detection in 1993 and 1994 of H6N8 (h6.2.1) there was an increase in diversity through time (last sampled in 2002; h6.2.4) to include association with other neuraminidase subtypes (N1, N2, N5, and N6). This pattern might be explained by the occurrence of reassortments during this time [50], [51]. Clade h6.2 was not sampled during our surveillance efforts, even though it is a North American clade. Therefore our surveillance results supported the conclusion that this lineage may have gone extinct through replacement by the Eurasian clade invasion into North America [50], [52].

H9 sequences from a public database combined with our surveillance sequences generated a phylogenetic tree with two major clades (Figure 9; h9.1 and h9.2). One major clade, h9.2, shared sequences from Eurasia and North America that included both wild and domestic birds. Clade h9.1 was primarily Asian H9N2 from domestic birds, with a small subclade including H9N2 from domestic turkeys and a chicken from the U.S. sampled in 1966 (h9.1.5). Our surveillance H9 sequences came from Nebraska, Oregon, and California, which are states unsampled in the public database. The addition of our surveillance sequences uncovered a novel clade of North American avian H9N2 sequences and appears to document a previously unidentified intercontinental relationship of the H9 subtype between Asia and the U.S. (h9.2.5). This newly identified subclade was sister to Asian H9N2 sequences from ducks sampled in Hong Kong in the late 1970’s. The localities where these U.S. sequences were collected suggested that an intercontinental transfer may have happened through the Pacific and/or Central flyway. The phylogeny documents at least three intercontinental transfer events of H9 in the last 40 years (h9.2.5, h9.2.6-h9.2.9, h9.2.10) and there was evidence of host exchange between wild birds and domestic birds within and between continents (h9.2.6-h9.2.9). Most of these H9 intercontinental transfer events have been documented previously [26], [27]. Recent work has shown that H9N2 frequently undergoes reassortments and changes pathogenicity and therefore has high potential to lead to novel pandemic viruses [53], [54]. Therefore, surveillance and monitoring of this subtype in North American birds is critical.

Phylogenetic analyses of two other subtypes, H13 and H16, also demonstrated intercontinental exchanges of avian influenza viruses. Phylogenetic relationships within both subtypes were documented only for wild birds based on available sequences. The H13 phylogeny had three major lineages (Figure 13; h13.1, h13.2, and h13.3), one exclusively North American (h13.2) and two in which North American sequences were mixed with Eurasian sequences (h13.1 and h13.3). Co-circulating diverse lineages of H13 were seen across continents [26], [27], [29], [55]. Wille et al. [55] found evidence of H13 virus genes in great black-backed gulls (*Larus marinus*) moving between North America and Europe, and provided additional evidence of actual movement by birds between these two continents based on band recovery data. Many of the assembled H13 sequences from North America (7 of 18) in our phylogeny were obtained from our surveillance effort, with our sequences forming a unique subclade within a previously identified uniquely North American H13 clade (h13.2.1) [H13g2 in [27], [29] (Figure 13). The seven H13N2 sequences contributed by this study increased the geographical range of this subtype in the U.S. to six new states including the southern U.S. (Alabama, Florida, Georgia, Mississippi, New York, and Ohio).

Based on our development of H16 specific primers [34] during the course of this study we were able to expand the known distribution of this subtype in the U.S (Figure 14; h16.1). Only one sequence meeting our criteria had been previously published from North America with the surveillance adding four new sequences from across the U.S. (h16.1.1). H16 was an infrequently sampled subtype across published sequences and in our surveillance, likely because this subtype is most common in gulls [7], [24], which have been sampled less frequently compared with waterfowl and shorebirds. However, even with this lower rate of sampling exchange between the U.S. and Scandinavia over the last 15 years is evident [24], [27], [29].

A lack of evidence for intercontinental exchange and high levels of genetic divergence between North American and Eurasian lineages were observed in the case of subtypes H4, H8, H10, H11, and H12. These HA subtypes are predominantly present in the U.S. with few Eurasian counterparts [26], [27], [52], a high degree of phylogenetic structure exhibiting a gradual evolution through time (e.g. the ladder-like structure of the North American H4 clade), and high levels of unique genetic diversity circulating among wild birds. Although geographic affinities are seen in some subclades, such as in the North American H10 where there seem to be affiliations among flyways (Figure 10), they are not strongly supported, suggesting that in the last 20 years some LPAIV HA subtypes have been circulating freely across the U.S. without input from Europe or Asia during that period.

H4 was the second most commonly sequenced subtype from the surveillance (N = 33, Figure 2). Overall, H4 sequences (75% of which were H4N6) showed a clear separation between North American (h4.1) and Eurasian (h4.2) wild bird HA sequences. Spackman et al. [21] also identified this genetic separation between the continents. We found three clusters of wild bird sequences within North America (Figure 6); one of which was exclusively composed of surveillance sequences (h4.1.1; N = 4). Therefore H4 may be primarily a wild bird virus and possibly less of an issue for poultry health. The addition of these sequences to other published avian North American samples from the past twenty years showed that we collected a number of samples representative of previously unsampled subclades and/or geographical localities (Figure 6) (Arizona, Colorado, Connecticut, Indiana, Louisiana, Maine, Michigan, Nebraska, New Mexico, North Dakota, Oklahoma, Pennsylvania, Washington, West Virginia, Wisconsin, Utah, Vermont).

Available H8 sequences were low in numbers overall (N = 12, Figure 2) with the H8N4 subtype most commonly represented (N = 10, Figure 8). Our surveillance effort detected only one H8 from Pennsylvania (h8.1.2). Previously this subtype had only been sequenced from samples collected on the west coast of the U.S. From the small number of complete H8 sequences assembled for our analysis, it appears there was a clade in North America (h8.1) that arose from an Asian source and was first detected in the U.S. in 1991. However, more recent exchange was not been detected.

Almost all of the H10 sequences we examined were from North American wild birds (N = 46 of 51). There were two major lineages in the H10 tree (Figure 10; h10.1 and h10.2), which demonstrate a lack of recent or regular intercontinental exchange. One lineage (h10.1) was primarily comprised of North American wild birds (except two sequence from domestic birds from California; h10.1.1) and with no Eurasian sequences. This clade showed a ladder-like progression of detection through time. The other lineage (h10.2) included five Eurasian ducks. The surveillance contributed three H10 sequences to the North American clade (h10.1.4) including one from Montana, where H10 sequences had not been reported in the past 20 years.

The H11 and H12 phylogenies formed clear continental separation with North American and Eurasian sequences forming separate reciprocally monophyletic groups (Figure 11, h11.1 and h11.2; Figure12, h12.1 and h12.2). North American H11 sequences, including isolates from the surveillance (h11.1.1) were associated with a diversity of neuraminidase subtypes. North American H12 subtypes were associated with N5 (h12.1), including three sequences from the surveillance (h12.1.1), with the exception of an H12N4 isolate from a laughing gull from Delaware (h12.1.2). North American H11 (Figure 11) showed some evidence of transfer between wild birds to chickens in the 1990’s (h11.1.1), while the H12 North American sequences were only from wild birds (h12.1). Our surveillance efforts added wild bird sequences from geographical localities that were not found in the publicly available sequence database from the past 20 years for both subtypes (H11: Montana, Oregon, Pennsylvania, South Dakota, Wisconsin; H12: Massachusetts, Montana, Nebraska). Further, the surveillance sequences formed unique subclades (h11.1.1).

### Wild Bird Subtype Genetic Diversity

Overall there was high genetic diversity within subtypes except in those that were represented by few samples (e.g. H8; Table 1). However we did not observe a significant correlation between subtype sample size and genetic diversity (*p*>0.4), showing that even relatively rare HA subtypes, such as H16 maintain a genetically diverse viral population under what appear to be relaxed selective constraints when compared to internal coding gene segments [56]. All subtypes across continents had virtually full haplotype diversity. Interestingly, our analysis of genetic differentiation within HA subtypes show significant differences between North American and Eurasia sequences (Table 1; H1, *K*_ST_ = 0.09449 to H12, *K*_ST_ = 0.40124 except H8, *K*_ST_ = 0.04479 and H16, *K*_ST_ = 0.05997), suggesting intercontinental exchange is relatively infrequent. For most subtypes there were more polymorphic sites in North American sequences than Eurasian sequences (except H8, H9, and H16). Individual sequences within HA subtypes showed a high degree of polymorphism (*θ*_W_ ranged from 0.07001 in H8 to 0.13663 in H16). The number of polymorphic sites across the sequenced gene ranged from 355 (H8) to 883 (H9) per subtype.

In some HA subtypes there were clear patterns of temporal divergences within North America (H1, H6, H10, and H13) and in the rest there was not a temporal element to the evolution of the subtype. We surveyed the assembled data sets for evidence of departure from neutral conditions of molecular evolution by employing a commonly used neutrality test statistic that draws information from the mutation frequency spectrum. In half of the examined subtypes (H1, H3, H4, H6, H9, H10, and H11) we found evidence for a recent population size expansion as manifested by an excess of rare substitutions. This was often driven by a significant signal of population expansion in North America that was not simultaneously found in Eurasia (H1, H4, H10, and H11). Our results suggested that many North American AIV subtypes reflected recent population expansion and subsequent increase in genetic diversity (Table 1). The phenomenon of detecting closely related AIV subtypes when sampling in the same timeframe or same locality was first identified in wild birds by Chen and Holmes [51] and demonstrates the rapid evolution that occurs within AIV subtypes. In fact, subtypes from our study that showed a signal of stable demographic trends should be tested after greater sampling (H2, H12, H13, and H16). Overall, we confirm what is known about AIV evolutionary dynamics [10], [51]. The evidence for inconstant intercontinental circulation of HA subtypes from the phylogenetic analysis combined with signs of population expansions resulting from antigenic shifts suggest that these viruses have a diverse array of adaptive behaviors that allow them to colonize new avian hosts in novel environments and allow for selective competition between subtypes or among lineages of the same subtype.

### Relevance to Influenza Surveillance in Wild Birds

A large-scale surveillance effort across the U.S. provided information that elucidated LPAIV HA genetic diversity and varying patterns of intercontinental (Eurasia to North America) viral exchange across subtypes. This surveillance effort uncovered previously unknown lineages in many subtypes including an H9 lineage with a previously unidentified exchange between Asian ducks (unknown if wild or domestic) and North American wild birds. Another significant contribution of the surveillance effort was that 8 of 12 AIV subtypes were detected in states where they had not been previously documented (in the NCBI Influenza Virus Resource). Also of importance is our increased understanding that diverse subtype lineages circulating across North America are unique to this continent. Although North America does not currently harbor Asian strain HPAIV H5N1, the rapid evolution that occurs within AIV subtypes warrants continued surveillance efforts. In addition, the sporadic intercontinental exchange of AIV strains suggests long-term, focused surveillance for AIV in wild birds is needed to detect infrequent introductions of novel AIV strains by wild birds that may affect agricultural and human health in the U.S.

Surveillance, particularly genetic surveillance, of AIV in wild birds has been recognized as a necessary component for detecting emerging global pandemic viruses relevant to humans and agricultural health [57], [58]. This study has shown that surveillance requires a large effort but that such an effort can be successful in sampling genetic diversity within and across subtypes that are co-circulating. Further, in combination with phylogenetic analyses, recent and historical transfer among continents can be identified. Subtypes that have been shown to experience multiple and recent intercontinental exchange can be monitored through surveillance efforts for potential reassortment and subsequent alteration to increased levels of pathogenicity. The wild bird fecal surveillance effort and subsequent phylogenetic analyses can now be used to strategically target geographical areas that are a high priority for surveillance in a continued effort to monitor AIV that may be of concern to humans, livestock, and poultry.

## Supporting Information

Table S1 **Internal primers designed to amplify H3, H6, H8, H9, and H13 subtypes.** *Internal primers were used in conjunction with HA external primers, Hgga+ and H-T7 as designed by SEPRL (Athens, GA),+ = forward, − = reverse.
(DOCX)

Table S2 **GenBank Accession numbers for each HA sequence generated during this study.**
(XLSX)

## References

[1] Webster RG, Bean WJ, Gorman OT, Chambers TM, Kawaoka Y (1992) Evolution and ecology of influenza A viruses. Microbiol Rev 56: 152–179. 1579108 10.1128/mr.56.1.152-179.1992 PMC372859

[2] Munster VJ, Baas C, Lexmond P, Waldenstrom J, Wallensten A, et al (2007) Spatial, temporal, and species variation in prevalence of influenza a viruses in wild migratory birds. PLoS Pathogens 3: 0630–0638. 10.1371/journal.ppat.0030061 PMC1876497 17500589

[3] Rohani P, Breban R, Stallknecht DE, Drake JM (2009) Environmental transmission of low pathogenicity avian influenza viruses and its implications for pathogen invasion. P Natl Acad Sci 106: 10365–10369. 10.1073/pnas.0809026106 PMC2690603 19497868

[4] Stallknecht DE, Brown JD (2007) Wild birds and the epidemiology of avian influenza. J Wildlife Dis 43: S15–S20.

[5] Stallknecht DE, Shane SM (1988) Host range of avian influenza virus in free-living birds. Vet Res Commun 12: 125–141. 3055662 10.1007/BF00362792

[6] Alexander DJ (2000) A review of avian influenza in different bird species. Vet Microbiol 74: 3–13. 10799774 10.1016/s0378-1135(00)00160-7

[7] Fouchier RAM, Munster V, Wallensten A, Bestebroer TM, Herfst S, et al (2005) Characterization of a novel influenza A virus hemagglutinin subtype (H16) obtained from black-headed gulls. J Virol 79: 2814–2822. 15709000 10.1128/JVI.79.5.2814-2822.2005 PMC548452

[8] Olsen B, Munster VJ, Wallensten A, Waldenstrom J, Osterhaus ADME, et al (2006) Global patterns of influenza A virus in wild birds. Science 312: 384–388. 16627734 10.1126/science.1122438

[9] Duan L, Campitelli L, Fan XH, Leung YHC, Vijaykrishna D, et al (2007) Characterization of low-pathogenic H5 subtype influenza viruses from Eurasia: Implications for the origin of highly pathogenic H5N1 viruses. J Virol 81: 7529–7539. 17507485 10.1128/JVI.00327-07 PMC1933357

[10] Chen H, Li Y, Li Z, Shi J, Shinya K, et al (2006) Properties and dissemination of H5N1 viruses isolated during an influenza outbreak in migratory waterfowl in western China. J Virol 80: 5976–5983. 16731936 10.1128/JVI.00110-06 PMC1472608

[11] Gilbert M, Xiao X, Domenech J, Lubroth J, Martin V, et al (2006) Anatidae migration in the western Palearctic and spread of highly pathogenic avian influenza H5NI virus. Emerg Infect Dis 12: 1650–1656. 17283613 10.3201/eid1211.060223 PMC3372333

[12] Koehler AV, Pearce JM, Flint PL, Franson JC, Ip HS (2008) Genetic evidence of intercontinental movement of avian influenza in a migratory bird: the northern pintail (*Anas acuta*). Mol Ecol 17: 4754–4762. 19140989 10.1111/j.1365-294X.2008.03953.x

[13] U.S. Interagency Working Group (2006) An early detection system for highly pathogenic H5N1 avian influenza in wild migratory birds: U.S. Interagency Strategic Plan. Washington, D.C.: U.S. Interagency Working Group.

[14] McLean RG, Hall JS, Franklin AB, Sullivan HJ, VanDalen KK, et al.. (2007) Avian influenza in wild birds: Environmental sampling for the rapid detection of avian influenza viruses. In: Notle DL, Arjo WM, Stalman DH, editors. 12th Wildlife Damage Management Conference.

[15] Deliberto TJ, Swafford SR, Nolte DL, Pedersen K, Lutman MW, et al (2009) Surveillance for highly pathogenic avian influenza in wild birds in the USA. Integr Zool 4: 426–439. 21392315 10.1111/j.1749-4877.2009.00180.x

[16] DeLiberto TJ, Swafford SR, Van Why K (2011) Development of a national early detection system for highly pathogenic avian influenza in wild birds in the United States of America. In: Majumdar S, Brenner F, Huffman J, McLean R, Panah A, editors. Pandemic Influenza Viruses: Science, Surveillance and Public Health. Easton, PA, USA: The Pennsylvania Academy of Science. 156–175.

[17] Schafer JR, Kawaoka Y, Bean WJ, Suss J, Senne D, et al (1993) Origin of the pandemic 1957 H2 influenza A virus and the persistence of its possible progenitors in the avian reservoir. Virology 194: 781–788. 7684877 10.1006/viro.1993.1319

[18] Makarova NV, Kaverin NV, Krauss S, Senne D, Webster RG (1999) Transmission of Eurasian avian H2 influenza virus to shorebirds in North America. J Gen Virol 80: 3167–3171. 10567648 10.1099/0022-1317-80-12-3167

[19] Liu J, Okazaki K, Bai G, Shi W, Mweene A, et al (2004) Interregional transmission of the internal protein genes of H2 influenza virus in migratory ducks from North America to Eurasia. Virus Genes 29: 81–86. 15215686 10.1023/B:VIRU.0000032791.26573.f1

[20] Widjaja L, Krauss SL, Webby RJ, Xie T, Webster RG (2004) Matrix gene of influenza A viruses isolated from wild aquatic birds: Ecology and emergence of influenza A viruses. J Virol 78: 8771–8779. 15280485 10.1128/JVI.78.16.8771-8779.2004 PMC479093

[21] Spackman E, Stallknecht DE, Slemons RD, Winker K, Suarez DL, et al (2005) Phylogenetic analyses of type A influenza genes in natural reservoir species in North America reveals genetic variation. Virus Res 114: 89–100. 16039745 10.1016/j.virusres.2005.05.013

[22] Wallensten A, Munster VJ, Elmberg J, Osterhaus ADME, Fouchier RAM, et al (2005) Multiple gene segment reassortment between Eurasian and American lineages of influenza a virus (H6N2) in Guillemot (*Uria aalge*). Rapid Communication. Arch Virol 150: 1685–1692. 15883657 10.1007/s00705-005-0543-8

[23] Glaser L, Zamarin D, Acland HM, Spackman E, Palese P, et al (2006) Sequence analysis and receptor specificity of the hemagglutinin of a recent influenza H2N2 virus isolated from chicken in North America. Glycoconjugate J 23: 93–99. 10.1007/s10719-006-5441-0 16575526

[24] Krauss S, Obert CA, Franks J, Walker D, Jones K, et al (2007) Influenza in migratory birds and evidence of limited intercontinental virus exchange. PLoS Pathogens 3: 1684–1693. 10.1371/journal.ppat.0030167 PMC2065878 17997603

[25] Dugan VG, Chen R, Spiro DJ, Sengamalay N, Zaborsky J, et al (2008) The evolutionary genetics and emergence of avian influenza viruses in wild birds. PLoS Pathogens 4: e1000076. 18516303 10.1371/journal.ppat.1000076 PMC2387073

[26] Liu S, Ji K, Chen J, Tai D, Jiang W, et al (2009) Panorama phylogenetic diversity and distribution of type A influenza virus. PLoS ONE 4: e5022. 19325912 10.1371/journal.pone.0005022 PMC2658884

[27] Shi W, Lei F, Zhu C, Sievers F, Higgins DG (2010) A complete analysis of HA and NA genes of influenza A viruses. PLoS ONE 5: e14454. 21209922 10.1371/journal.pone.0014454 PMC3012125

[28] Lee D-H, Lee H-J, Lee Y-N, Park J-K, Lim T-H, et al (2011) Evidence of intercontinental transfer of North American lineage avian influenza virus into Korea. Infect Genet Evol 11: 232–236. 20933610 10.1016/j.meegid.2010.09.012

[29] Wille M, Robertson GJ, Whitney H, Bishop MA, Runstadler JA, et al (2011) Extensive geographic mosaicism in avian influenza viruses from gulls in the northern hemisphere. PLoS ONE 6: e20664. 21697989 10.1371/journal.pone.0020664 PMC3115932

[30] Farnsworth ML, Kendell WL, Doherty PF, Miller RS, White GC, et al. (2011) Targeted surveillance for highly pathogenic avian influenza in migratory waterfowl across the conterminous United States. In: Majumdar SK, Brenner FJ, Huffman JE, McLean RG, Panah AI et al.., editors. Pandemic influenza viruses: Science, surveillance and public health. Easton, PA, USA: The Pennsylvania Academy of Science.

[31] Spackman E, Senne DA, Myers TJ, Bulaga LL, Garber LP, et al (2002) Development of a real-time reverse transcriptase PCR assay for type A influenza virus and the avian H5 and H7 hemagglutinin subtypes. J Clin Microbiol 40: 3256–3260. 12202562 10.1128/JCM.40.9.3256-3260.2002 PMC130722

[32] VanDalen KK, Franklin AB, Mooers NL, Sullivan HJ, Shriner SA (2010) Shedding light on avian influenza H4N6 infection in mallards: Modes of transmission and implications for surveillance. PLoS ONE 5: e12851. 20877466 10.1371/journal.pone.0012851 PMC2942899

[33] Szretter KJ, Balish AL, Katz JM (2006) Influenza: Propagation, quantification, and storage. Current Protocols in Microbiology: John Wiley & Sons, Inc. 10.1002/0471729256.mc15g01s3 18770580

[34] VanDalen K, Anderson T, Killian M, Pedersen J, Franklin A, et al (2008) Increased detection of influenza A H16 in the United States. Arch Virol 153: 1981–1983. 18825483 10.1007/s00705-008-0213-8

[35] Suarez DL, Perdue ML, Cox N, Rowe T, Bender C, et al (1998) Comparisons of highly virulent H5N1 influenza A viruses isolated from humans and chickens from Hong Kong. J Virol 72: 6678–6688. 9658115 10.1128/jvi.72.8.6678-6688.1998 PMC109865

[36] Horimoto T, Rivera E, Pearson J, Senne D, Krauss S, et al (1995) Origin and Molecular Changes Associated with Emergence of a Highly Pathogenic H5N2 Influenza Virus in Mexico. Virology 213: 223–230. 7483266 10.1006/viro.1995.1562

[37] Watterson GA (1975) On the number of segregating sites in genetical models without recombination. Theor Popul Biol 7: 256–276. 1145509 10.1016/0040-5809(75)90020-9

[38] Nei M (1987) Molecular evolutionary genetics. New York: Columbia University Press. 448 p.

[39] Fu YX (1997) Statistical tests of neutrality of mutations against population growth, hitchhiking and background selection. Genetics 147: 915–925. 9335623 10.1093/genetics/147.2.915 PMC1208208

[40] Ramos-Onsins SE, Rozas J (2002) Statistical properties of new neutrality tests against population growth. Mol Biol Evol 19: 2092–2100. 12446801 10.1093/oxfordjournals.molbev.a004034

[41] Hudson R (1990) Gene genealogies and the coalescent. In: Futuyma DJ, Antonovics J, editors. Oxford Surveys in Evolutionary Biology: Oxford Univ. Press, Oxford.

[42] Hudson RR, Boos DD, Kaplan NL (1992) A statistical test for detecting geographic subdivision. Mol Biol Evol 9: 138–151. 1552836 10.1093/oxfordjournals.molbev.a040703

[43] Librado P, Rozas J (2009) DnaSP v5: a software for comprehensive analysis of DNA polymorphism data. Bioinformatics 25: 1451–1452. 19346325 10.1093/bioinformatics/btp187

[44] Wessa P (2011) Free statistics software, Office for Research Development and Education, version 1.1.23-r7.

[45] Stamatakis A (2006) RAxML-VI-HPC: maximum likelihood-based phylogenetic analyses with thousands of taxa and mixed models. Bioinformatics 22: 2688–2690. 16928733 10.1093/bioinformatics/btl446

[46] Stamatakis A, Ott M (2008) Exploiting fine-grained parallelism in the phylogenetic likelihood function with MPI, Pthreads, and OpenMP: A performance study. In: Chetty M, Ngom A, Ahmad S, editors. Pattern Recognition in Bioinformatics: Springer Berlin/Heidelberg. 424–435.

[47] Lanave C, Preparata G, Sacone C, Serio G (1984) A new method for calculating evolutionary substitution rates. J Mol Evol 20: 86–93. 6429346 10.1007/BF02101990

[48] Yang Z (1994) Maximum likelihood phylogenetic estimation from DNA sequences with variable rates over sites: Approximate methods. J Mol Evol 39: 306–314. 7932792 10.1007/BF00160154

[49] Felsenstein J (1985) Confidence limits on phylogenies: An approach using the bootstrap. Evolution 39: 783–791. 28561359 10.1111/j.1558-5646.1985.tb00420.x

[50] Zu DH, Li J, Cardona CJ, Miller J, T.E C (2009) Invasions by Eurasian avian influenza virus H6 genes and replacement of its North American clade. Emerg Infect Dis 15: 1040–1045. 19624918 10.3201/eid1507.090245 PMC2744232

[51] Chen R, Holmes EC (2009) Frequent inter-species transmission and geographic subdivision in avian influenza viruses from wild birds. Virology 383: 156–161. 19000628 10.1016/j.virol.2008.10.015 PMC2633721

[52] Bahl J, Vijaykrishna D, Holmes EC, Smith GJD, Guan Y (2009) Gene flow and competitive exclusion of avian influenza A virus in natural reservoir hosts. Virology 390: 289–297. 19501380 10.1016/j.virol.2009.05.002 PMC2753668

[53] Alexander PE, De P, Rave S (2008) Is H9N2 avian influenza virus a pandemic potential? Can J Infect Dis Med 20: e35–336. 10.1155/2009/578179 PMC2706408 20514156

[54] Bi Y, Lu L, Li J, Yin Y, Zhang Y, et al (2011) Novel genetic reassortants in H9N2 influenza A viruses and their diverse pathogenicity to mice. Virol J 8: 505. 22050764 10.1186/1743-422X-8-505 PMC3236014

[55] Wille M, Robertson G, Whitney H, Ojkic D, Lang A (2011) Reassortment of American and Eurasian genes in an influenza A virus isolated from a great black-backed gull (*Larus marinus*), a species demonstrated to move between these regions. Arch Virol 156: 107–115. 21053031 10.1007/s00705-010-0839-1

[56] Obenauer JC, Denson J, Mehta PK, Su X, Mukatira S, et al (2006) Large-scale sequence analysis of avian influenza isolates. Science 311: 1576–1580. 16439620 10.1126/science.1121586

[57] Stöhr K (2003) The global agenda on influenza surveillance and control. Vaccine 21: 1744–1748. 12686087 10.1016/s0264-410x(03)00065-3

[58] Escorcia M, Juarez Estrada M, Attene-Ramos M, Nava G (2012) Improving global influenza surveillance: trends of A(H5N1) virus in Africa and Asia. BMC Res Notes 5: 62. 22268987 10.1186/1756-0500-5-62 PMC3305413

