# Supplementary material for: Molecular Surveillance of Low Pathogenic Avian Influenza Viruses in Wild Birds across the United States: Inferences from the Hemagglutinin Gene
Source: PLoS One. 2012 Dec 4;7(12):e50834. doi: 10.1371/journal.pone.0050834 (PMC3514193; doi:10.1371/journal.pone.0050834)
Supplement: Table S1 — Internal primers designed to amplify H3, H6, H8, H9, and H13 subtypes. *Internal primers were used in conjunction with HA external primers, Hgga+ and H-T7 as designed by SEPRL (Athens, GA),+ = forward, − = reverse. (DOCX) [file pone.0050834.s001.docx]

Table S1. Internal primers designed to amplify H3, H6, H8, H9, and H13 subtypes.

| Primer Name* | Primer Sequence (5’-3’) |
| --- | --- |
| H3+720 | TTGGGTCAGAGGCCAATCAGG |
| H3+851 | GCTCRATAATGAGATCAGATGCAC |
| H3-1070 | AAYCCTGCTATTGCACCGAACAG |
| H3-1159 | TAAGGTCTGCSGCTTGTCCTG |
| H6+668 | TGAATTTYGCCAAGAGTCCGG |
| H6+894 | GACTATTGCAGGAGTCCTAAGG |
| H6-1090 | CTATCATTCCAGTCCAYCCTCC |
| H6-1244 | AATTCRTGGTCGACAGCTTC |
| H8-1073 | CAGCAATGGCTCCGAATAGC |
| H8-1165 | CTGGTCAGCTGCCATTCCTG |
| H8+872 | GAGGACATACCCATCGGAAACTG |
| H8+728 | AGAGGACARCAAGGAAGAATGG |
| H9+598 | GGCATACACCATCCACCTACTG |
| H9+841 | GGAGAAAGCCATGGAAGAATC |
| H9-1171 | GYGTTGATTCCCTYTCTGCAGCC |
| H9-1085 | CCTCCTTCTATGAATCCAGC |
| H13+740 | CCTGGGTACAATGGTCAAAGGAG |
| H13+827 | GGATTGTTGGCCCCAAGATATGG |
| H13-1117 | CCTGGCCAACCACCT TCTATG |
| H13-1347 | GACCACACATCAGTCACAGC |
